# Using Nanobodies to Study Protein Function in Developing Organisms

**DOI:** 10.3390/antib8010016

**Published:** 2019-02-12

**Authors:** Gustavo Aguilar, Shinya Matsuda, M. Alessandra Vigano, Markus Affolter

**Affiliations:** Biozentrum, University of Basel, Klingelbergstrasse 70, 4056 Basel, Switzerland; gustavo.aguilar@unibas.ch (G.A.); shinya.matsuda@unibas.ch (S.M.); alessandra.vigano@unibas.ch (M.A.V.)

**Keywords:** nanobody, GFP, *C. elegans*, development, drosophila, zebrafish

## Abstract

Polyclonal and monoclonal antibodies have been invaluable tools to study proteins over the past decades. While indispensable for most biological studies including developmental biology, antibodies have been used mostly in fixed tissues or as binding reagents in the extracellular milieu. For functional studies and for clinical applications, antibodies have been functionalized by covalently fusing them to heterologous partners (i.e., chemicals, proteins or other moieties). Such functionalized antibodies have been less widely used in developmental biology studies. In the past few years, the discovery and application of small functional binding fragments derived from single-chain antibodies, so-called nanobodies, has resulted in novel approaches to study proteins during the development of multicellular animals in vivo. Expression of functionalized nanobody fusions from integrated transgenes allows manipulating proteins of interest in the extracellular and the intracellular milieu in a tissue- and time-dependent manner in an unprecedented manner. Here, we describe how nanobodies have been used in the field of developmental biology and look into the future to imagine how else nanobody-based reagents could be further developed to study the proteome in living organisms.

## 1. Introduction

Antibodies have been invaluable tools in basic biological sciences for several decades. Polyclonal and monoclonal antibodies can be used for manifold studies, for example, to detect the amount of a given protein in Western blots, to isolate proteins or protein complexes from cell lysates, or to localize proteins in fixed cells and tissues, just to name a few applications. More rarely, antibodies have been used for functional studies in cultured cells or in developing organisms, in particular via injection either into cells, into the body cavity of developing organisms, or into the blood stream of developing or adult organisms. The large size and multi-domain nature of antibodies as well as their instability in the intracellular milieu has hindered their more widespread use to manipulate protein function in vivo.

A major breakthrough in antibody research was made when Hamers and colleagues reported in 1993 that camels produce, in addition to the well-characterized antibodies containing a heavy and a light chain, an additional and distinct species of antibodies containing only a heavy chain [1]. The binding affinity and selectivity of these single-chain antibodies turn out to be comparable to that of classical antibodies, and the high-affinity antigen-recognizing region can be isolated from the single heavy chain and expressed as a single polypeptide chain. Such small antibody fragments were called VHH (from variable domain of heavy chain antibodies, also referred to as nanobodies) and they have dramatically changed the way antibodies have been used in developmental biology (and in many other research fields, such as structural biology, super resolution microscopy, etc., see other articles in this issue). 

## 2. From Cultured Cells to Developing Organisms

The function and the behavior of a cell is to a large extent determined by its proteome, i.e., by the proteins that are expressed in a given cell as a function of complex transcriptional regulation in the nucleus. While CRISPR/Cas technologies have paved the way to fast, cheap, and efficient genome editing [2], it remains more difficult to acutely manipulate proteins in a direct and desired manner in time and space. Recognition of DNA sequences via the injection or expression of the CRISPR/Cas module is highly efficient and selective due to guide RNAs providing target site selectivity. In contrast, recognition of proteins by classical antibodies in the intracellular milieu is often inefficient since antibodies consist of four chains that have to be properly assembled and folded and then kept in a stable configuration via disulfide bridges. The small size of single-chain antibody fragments and the ease to express them from integrated transgenes in cells has opened up the route to many novel applications in the complex context of multicellular organisms. In this article, we summarize studies in the field of developmental biology that have made use of nanobodies in multicellular animals and refer the reader to excellent recent reviews summarizing studies done in cultured cells [3,4,5,6]. Several developmental studies have used protein binders other than nanobodies, including single-chain antibodies (scFvs), Designed Ankyrin Repeat Proteins (DARPins) [7] monobodies [8], and others; we will not describe these studies here, but refer the reader to recent reviews on the topic [9,10].

## 3. Use of Functionalized Nanobodies in Multicellular Animals in the Context of Developmental Biology Studies

While antibodies and nanobodies have often been used to mask (and thereby inhibit) a functional domain of a protein of interest (POI) [11,12], the availability of the easily cloneable binding domains of nanobodies has stimulated researchers to generate transgenes that link this binding region to specific functional domains. This allows for the expression of protein fusions that are not only able to associate with the POI, but also to manipulate its function in a desired manner. First reports to do so using the nanobody scaffold involved the fusion of a fluorescent protein to a nanobody recognizing a POI, thereby visualizing the POI within cells (such nanobody fusions are also referred to as chromobodies, see below). Further experimental setups included the fusion of degradation-inducing domains, localization domains, and enzymatic domains to nanobodies. In the next sections, we highlight studies done in multicellular model organisms using nanobodies. Rather than describing in detail the biological findings resulting from these studies, we describe the generation and application of different nanobody-based tools and how they allow to manipulate and study protein function. 

### 3.1. Protein Degradation

A straightforward approach to studying the function of a protein during development is to remove it from a given cell population and investigate the molecular, developmental or physiological consequences of its absence. In most cases, such studies have been done indirectly using forward and reverse genetic approaches, as well as tissue-specific genetic manipulations in more recent years (using site-specific recombination or RNA interference) [13]. Another way to remove a POI is to target it for proteasomal degradation. To achieve this, a nanobody can be fused to a subunit of the E3 ubiquitin ligase complex (there are several protein domains such as F-boxes or SOCS-boxes that characterize such E3 ligases), which ultimately results in the recruitment of the POI to the complex, polyubiquitination of the nanobody-binding POI and its subsequent degradation via the proteasome. This approach was first reported by Kuo and colleagues [14] in cell culture and by Caussinus and colleagues (called deGradFP), who used it to degrade proteins in living drosophila embryos and larvae [15,16]. Caussinus et al. [17] made use of a nanobody which recognizes the Green Fluorescent Protein (GFP) and fused it to the N-terminal F-box domain of the drosophila Slmb protein, an adaptor protein which is part of the E3 ubiquitin ligase complex SCF and is required to mediate substrate-specific ubiquitination. This functionalization of the GFP nanobody allows GFP-, Venus- and YFP-tagged proteins to be recognized by the SCF complex and to be targeted for degradation. 

deGradFP has been used in a number of different studies in drosophila to address, for example, the role of actomyosin during tissue morphogenesis [18,19,20,21], to study the function of proteins in adult memory function and maintenance [22], to degrade POIs and study their contribution to Septate Junction establishment and maintenance [23], to analyze the role of the tissue-specificity of Hox gene function [24] and the role of certain proteins during microtubule network remodeling [25].

deGradFP has been used mostly in drosophila and was also shown to be able to induce protein degradation in mammalian cells [16]. However, as it turned out more recently, expression of the Slmb F-box to the GFP nanobody does not result in very efficient degradation of GFP fusion proteins when assayed in zebrafish embryos [26]. Based on this finding, two groups have further developed the method. In one case, the GFP nanobody was fused to an auxin-induced degron (AID), which was then shown to allow efficient and reversible degradation of GFP fusion proteins in zebrafish embryos upon addition of auxin [27]. In another study, the F-box of drosophila Slmb in the deGradFP fusion protein was replaced with the homologous sequence from zebrafish Slmb to generate a system called zGrad [26]. Using tissue-specific and inducible promoters in combination with functional GFP fusion proteins, it was shown that zGrad can induce the degradation of transmembrane, cytosolic, and nuclear proteins globally, locally, and temporally-controlled in different zebrafish tissues, and that such protein degradation can generate loss-of-function phenotypes. A system for protein degradation similar to deGradFP was also developed for *Caenorhabditis elegans*. In this particular case, the GFP nanobody was fused to a SOCS-box containing ubiquitin ligase adaptor in order to target GFP-tagged proteins for degradation [28]. To deplete a POI, GFP was either inserted into the endogenous locus of interest using CRISPR-Cas9 or via the rescue of a null mutant with a GFP fusion construct. This approach allowed for efficient tissue-specific protein ablation in *C. elegans* [29,30,31,32].

Several similar strategies have been reported and used in the last few years to induce degradation of specific POIs. Shin et al. [33] reported that the fusion of the GFP nanobody to a portion of SPOP (Speckle-type POZ-domain protein), a E3 ligase adaptor protein based on Cullin 3 acting in the nucleus, can induce exclusive nuclear degradation of GFP-tagged proteins in zebrafish embryos. This is an interesting addition to the other nanobody-based degradation methods, since it targets only the nuclear fraction of a POI. 

As more and more lines expressing endogenously-tagged fluorescent proteins are becoming available in the different model systems due to the widespread use of Crispr/Cas9-based genome editing technologies, these degradation systems will become extremely useful new additions to the existing toolbox for the analyses of protein function in complex multicellular animals. The advantage of using protein degradation in contrast to classical genetic approaches to study the consequences of depleting a POI are several-fold. First, mRNA and proteins might be delivered by the mother into the egg, in which case zygotic loss of function genetic analyses are complicated by the prevailing maternal contribution. As shown by several studies, such maternal proteins can efficiently be degraded by deGradFP and zGrad [34,35]. In other cases, the use of tissue-specific and/or inducible drivers expressing the nanobody-F-box chimera can lead to tissue-specific and inducible protein degradation, respectively, and allows to study a subset of functions of a POI. Alternatively, proteins might be very stable and persist for extended periods of time, despite the removal of the gene or the mRNA under study. This is particularly important to keep in mind for studies in adult organisms, in which many proteins might be rather stable and do not dilute out by cell division. Interestingly, expression of nanobody-ubiquitin ligase adaptor fusions can be controlled by temperature-controlled promoters, thus allowing reversible expression and recovery of protein levels in adult flies, as pioneered by the Hugo Bellen’s lab [22], and it is to be expected that many more studies of this type will be reported in the near future. 

### 3.2. Protein Relocalization and Trapping

Many proteins function in distinct cellular compartments (nucleus, cytoplasm, etc.) or are linked to specific cellular structures (different membrane compartments, surface of different organelles). To investigate the role of such distinct localization, nanobodies have proven to be extremely useful in altering the localization of POIs and investigate the consequences thereof. 

In a system called GrabFP, Harmansa et al. [36] constructed three nanobody-based GFP traps that localize to defined regions along the apico-basal axis of epithelial cells in drosophila. By fusing the GFP nanobody to a transmembrane domain such that the nanobody moiety is either exposed to the extracellular or to the intracellular milieu, the different GrabFP constructs allow to trap or localize proteins to distinct apico-basal positions and ask what developmental and molecular consequences this might have. GrabFP has been used to study myosin activation via Yorkie localization at the junctional cortex [37], to better define the role of Dishevelled activity in maintaining planar polarity complexes in epithelial tissues [38], the role of Dpp/Bone morphogenetic protein 2/4 dispersal in the basolateral compartment of the wing imaginal disc in drosophila [36], and to study the importance of plasma membrane location of apoptotic caspases for non-apoptotic functions [39]. In addition to this, transmembrane scaffolds, a lipid binding domain (PH domain) has also been proposed as membrane-tether for nanobody functionalization [40].

Such relocalization or trapping experiments might be particularly interesting when it comes to study secreted molecules that depend on their dispersal in in vivo settings. Secreted signaling molecules such as morphogens or hormones play crucial roles in animal development [41]. Being able to interfere with the extracellular distribution of such molecules in a predictable manner might allow to better understand the way and the importance of their dispersal in complex tissues. A system called Morphotrap, consisting of a GFP-binding nanobody fused to a transmembrane domain exposed on the surface of expressing cells, has been used to trap the GFP-fused ligand Dpp/Bmp2 in drosophila imaginal discs [42], to trap secreted GFP-fused Wnt in *C. elegans* [43] and to trap GFP-fusion proteins of the Nodal family in zebrafish [44]. Such studies allow to investigate the requirement for dispersal of secreted signaling molecules in vivo. Furthermore, these methods provide a means to generate gradients of different shapes across tissues and to investigate the developmental consequences of such altered gradients.

This toolbox has been recently been further expanded by the addition of a low affinity Morphotrap version [45]. In this case, the authors exchanged the previously used high-affinity GFP nanobody [17] with a low-affinity GFP nanobody [46]. This new tool allowed to finely tune extracellular GFP diffusivity in living zebrafish embryos.

In addition to the relocalization or trapping of proteins using nanobodies, Janusche and colleagues [47] made use of the modular nature of protein domains and combined the MS2 system and nanobody expression to alter the subcellular localization of mRNA molecules in drosophila neuroblasts. In this particular case, the mRNA was tagged with GFP using the MS2 system [48], while the nanobody against GFP was fused to specific subcellular localization domains, resulting in the efficient mislocalization of the GFP-decorated mRNA molecules. 

### 3.3. Protein Post-Translational Modification

Several enzymes (for example kinases) have many different substrates in a given cell or in different cells during development, and it remains rather challenging to unravel the complexity of such complex networks. Nanobodies can be used to direct enzymes to specific and unique substrates through direct protein–protein interaction and thereby lead to enhanced target specificity. In a proof-of-principle study, Roubinet et al. [49] fused the constitutively active minimal kinase domain of Rho kinase to a GFP-binding nanobody and an apical localization domain. Co-expression of this fusion construct together with GFP-myosin regulatory light chain in drosophila neuroblasts resulted in the ectopic accumulation of the phosphorylated form of the myosin light chain in the apical cytoplasmic compartment. In case this approach would work well with other enzymes, it would certainly contribute to a better understanding of complex regulatory circuits in developing organisms.

### 3.4. Protein Visualization

A very interesting application of nanobodies is the visualization of endogenous proteins in living organisms using fluorescently labelled nanobodies (also referred to as chromobodies; [17]). Chromobodies as ready-to-use tools in developmental biology might be particularly useful if they are directed against proteins of general interest or against proteins that mark different cellular compartments or cell states. Rothbauer and colleagues have generated chromobodies against the major cytoskeletal component Actin and the cell cycle marker PCNA and validated their use in zebrafish embryos [50]. For this purpose, nanobodies binding directly to these proteins were isolated from camelids [51,52]. Chromobodies should be built from binders directed against functionally inert epitopes such as to avoid unwanted effects on mobility and function of the POI, and thus have to be carefully selected and validated for each POI. Due to the usefulness for a wide community of biologists, such chromobody-expressing transgenic animals will most likely become reliable and important additions for future developmental studies. 

When proteins are tagged endogenously with GFP, distinguishing protein dynamics of a single cell can be difficult in crowded tissues where neighbor cells also express the tagged protein. To achieve single neuron protein dynamics, Kamiyama et al. [53] designed a chromobody against GFP that, when expressed in particular neurons by tissue-specific promoters or expression systems, was able to mark the GFP-fused POI with a red fluorescent protein, and thereby differentially label these neurons from the neighboring cells.

Given the highly dynamic protein expression in the developing drosophila embryo, some proteins are degraded faster than the fluorescent tag matures (up to >30 min for GFP in vivo), impeding protein visualization via this method. To solve this problem, a different tool has been designed, the LlamaTags. Instead of fusing the POI to GFP, the POI was fused to a nanobody recognizing GFP, and GFP itself was used as soluble cytoplasmic substrate that follows the POI by binding to it; while GFP itself distributed in the cytoplasm, the expression of the nanobody-fused transcription factor resulted in the nuclear translocation of GFP [54]. Following the same concept, mCherry nanobodies and soluble mCherry allowed to perform multicolor visualization of protein dynamics. Using LlamaTag, the dynamics of transcription factors in the early drosophila embryo was followed in time and space, allowing unprecedented insight into the mechanisms of coordinated gene expression in these syncytial embryos.

### 3.5. Protein Scaffolding and Cell–Cell Contact Reporters

Nanobodies have also been used in developmental studies in a more synthetic approach, allowing to trigger certain functions when a scaffolding protein is present. This is achieved in the cell of interest via the use of two distinct binders recognizing a scaffold in a non-overlapping fashion, bringing two different components to the same scaffold complex. The Cepko laboratory has used two GFP-binding nanobodies to assemble different activities in only those cells that express GFP. In a method called “transcription device dependent of GFP” (T-DDOG; [55]), both a DNA-binding domain and a transcription regulatory domain (resulting in activation or repression) was fused to one and the other GFP binder, respectively; these two activities are only assembled into one protein complex in those cells that express GFP, thus allowing to target the activation or repression of desired genes to those particular cells. This method was used to regulate gene expression in both mice and zebrafish. The same approach has also been used to reconstitute a split Cre recombinase [56], allowing to make recombination dependent on the presence of GFP, and can be adopted for many more applications.

While T-DDOG exploits the presence of an intracellular GFP to trigger a response, others have designed receptors to elicit transgene activation upon recognition of extracellular antigen in other cells. To achieve that, the Notch receptor was engineered by replacing the extracellular region with a protein binder and the intracellular tail by a transcriptional activator. Upon recognition of the extracellular antigen, the intracellular domain is cleaved, and the C-terminal transcriptional activator is thereby released to translocate to the nucleus and activate transgene expression [57]. This concept has been used in developing drosophila embryos to trace cell–cell contacts between cells expressing membrane-bound GFP and cells containing synthetic Notch receptors exposing GFP nanobody on the cell surface as well as a transgene expressing a fluorescent label upon activation of this synthetic Notch receptor [57].

## 4. A look Into the Future

With the exception of chromobody applications, one striking aspect of all the nanobody-based developmental studies described above is that they have relied on the almost exclusive use of one or two nanobodies binding to GFP. These nanobodies have been isolated from camels upon immunization with GFP and were well characterized in vitro and in vivo [58,59]. Since GFP was discovered [60] and its sequence cloned [61], it has been extensively used as a fusion partner to follow protein expression and dynamics in vivo. The many transgenic GFP lines available in the different model systems make the use of validated GFP nanobody-based tools rather straightforward. 

Probably the most favored applications of antibodies in biomedical research is their use as blocking reagents, binding with high affinity to an active site or a site involved in essential protein–protein interactions and hence interfering with protein function upon binding. This approach has not been used much thus far in developmental biology, because nanobodies against endogenous proteins of model organisms such as *C. elegans*, drosophila, or zebrafish have not been identified and reported, with the exception of the nanobodies used in the context of chromobodies. In the last few years, several labs have generated and studied nanobodies directed against cellular proteins [5], but most of the studies reported so far have targeted human proteins and have been studied in cultured cells. It will be very interesting to use these or similar nanobodies in multicellular animals to dissect cell biological processes in vivo. However, the ease with which GFP binders can be used across species is most likely not replicated by these nanobodies isolated against cellular proteins, since it is unlikely that many nanobodies isolated against human proteins will recognize the homologous protein from *C. elegans* or drosophila. Nevertheless, the availability of recently described screening devices [62] or platforms will speed up the isolation and characterization of nanobodies against endogenous proteins in different model organisms used in the field of developmental biology. Therefore, it can be expected that nanobodies binding endogenous POIs will be used more often in the future, especially in species, in which tools for efficient genetic manipulation are less common. They might be used in the context of similar functionalization as already described (degradation, relocalization, chromobodies, etc.), to mask the function of a protein or certain subdomains thereof, to detect or interfere with post-translational modifications, or even as reagents stabilizing specific protein conformations, as already proposed for some nanobodies in cell culture [62]. In each case, however, the specificity of the nanobody has to be carefully evaluated in the context of the developmental system used.

One of the obvious limitations of the use of the GFP nanobodies is the failure to endogenously tag certain proteins with GFP due to functional interference. Recently, nanobodies that are able to bind to short linear epitopes have been isolated [63,64]. Upon the insertion of such a tag at the endogenous locus of a protein to be studied, it should be possible to manipulate the latter with the corresponding functionalized binder, thereby bypassing the isolation and validation procedures involved in obtaining POI-specific nanobodies. Other binders, such as single-chain antibodies derived from IgGs, have been shown to bind short epitopes; however, the multidomain structure of these binders is normally far from ideal in the cell cytoplasm (with some exceptions, [65,66]). Since binders against small epitopes can more easily be validated in complex multicellular animals (by showing that they do not influence developmental processes in the absence of the epitope tag), and since endogenous gene tagging has become very efficient using Crisp/Cas, such binders will probably be used extensively to manipulate protein function intracellularly in combination with the available functionalization domains.

**Figure 1:**
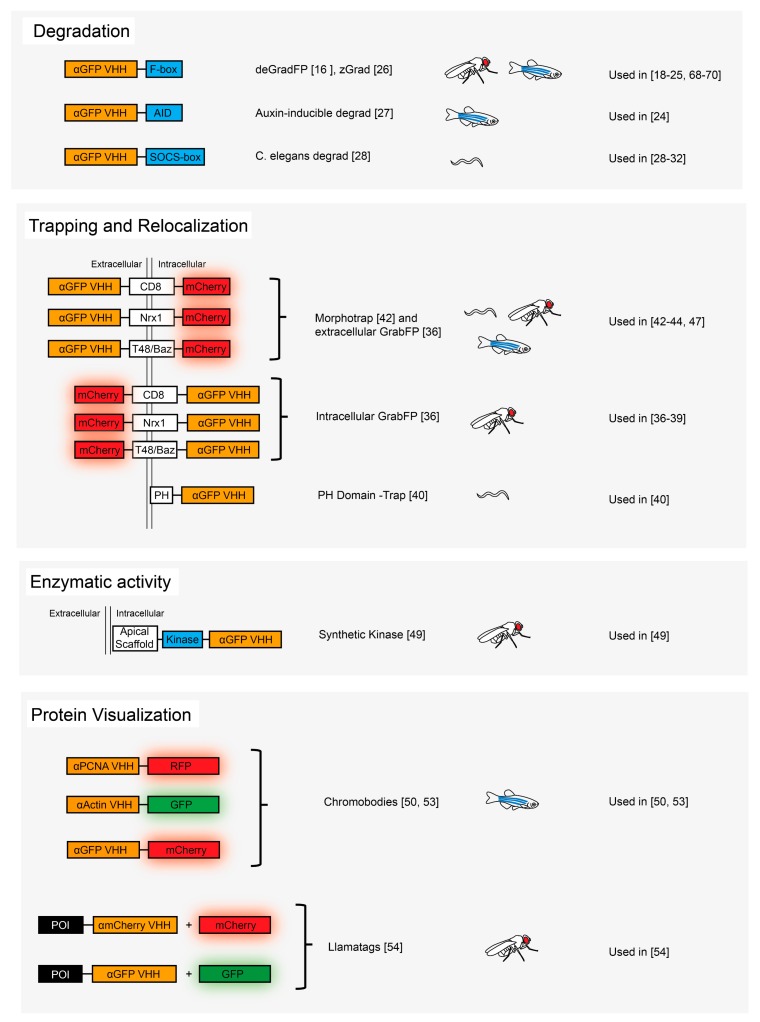
Different nanobody-based methods applied to developmental biology [16,18,19,20,21,22,23,24,25,26,27,28,29,30,31,32,36,37,38,39,40,42,43,44,47,49,50,53,54,68,69,70].

One of the most exciting aspects of the use of nanobodies in developmental biology is that they can be fused to functionalization domains to generate novel reagents which specifically and directly target a POI and manipulate it in a given, desired manner (see Figure 1). The list of such potential functionalization is long, but it is likely that many possible manipulations have not even been thought of in the early days of these novel possibilities to investigate the proteome. The emergence of more and more complex functionalization strategies requires a tight control of their performances to avoid undesired effects. The development of nanobodies that promote their own degradation when the POI is not present [67] is among the incipient strategies to achieve this tight control. It will be interesting to follow how the use of nanobody-based tools will evolve in the future, and it is hoped that this approach, combined with many other approaches (such as optogenetics), will allow to better understand the role of the proteome in development and disease.
